# A cross‑sectional analysis of vitamin D status and associated factors in children recovering from severe acute malnutrition in Zambia and Zimbabwe

**DOI:** 10.1371/journal.pone.0355404

**Published:** 2026-08-28

**Authors:** Tracy N. Phiri, Cherlynn Dumbura, Kuda Mutasa, Deophine Ngosa, Nivea Chuulu, Florence D. Majo, Sandra Rukobo, Claire D. Bourke, Beatrice Amadi, Mutsa Bwakura-Dangarembizi, Adrian R. Martineau, Andrew J. Prendergast, Paul Kelly

**Affiliations:** 1 Tropical Gastroenterology & Nutrition group, University of Zambia School of Medicine, Lusaka, Zambia; 2 Centre for Immunobiology, University of Glasgow College of Medical Veterinary and Life Sciences, Glasgow, United Kingdom; 3 Institute for Maternal and Child Health Research, Harare, Harare Province, Zimbabwe; 4 Blizard Institute, Barts & The London School of Medicine and Dentistry, Queen Mary University of London, London, United Kingdom; PLOS: Public Library of Science, ETHIOPIA

## Abstract

Mortality following severe acute malnutrition (SAM) remains high in Africa. Some children with SAM have altered immune responses and impaired neurodevelopment; however, whether vitamin D deficiency contributes to these poor outcomes remains unclear. We conducted a cross-sectional analysis of circulating 25-hydroxyvitamin D [25(OH)D] concentrations at hospital discharge (25(OH)D) in 442 children aged 0–59 months with SAM at one hospital in Lusaka, Zambia, and two hospitals in Harare, Zimbabwe. Also, we investigated the relationship between 25(OH)D concentrations and host factors. In 442 children, plasma 25(OH)D concentrations were measured using ELISA. Vitamin D deficiency was identified in 33/442 (7.5%; 95% CI: 5.2–10.3) children, all of whom were from Zimbabwe. A multivariable regression model that included age, sex, HIV status, season, oedema, cerebral palsy, and country demonstrated significantly higher 25(OH)D levels among children aged 6–11 months (adjusted (adj) ratio; 1.3; 95% CI: 1.1, 1.6; P = 0.003), 12–23 months (adj ratio; 1.5; 95% CI: 1.3, 1.8; P < 0.001), and 24–59 months (adj ratio; 1.5; 95% CI: 1.3, 1.8; P < 0.001) compared with those <6 months. Sampling during the cool season (adj ratio; 1.2; 95% CI: 1.1, 1.3; P = 0.002), absence of cerebral palsy (adj ratio; 1.2; 95% CI: 1.1, 1.4; P = 0.002) and residence in Zambia (adj ratio; 1.2; 95% CI: 1.1, 1.2; P < 0.001) were associated with higher concentrations of 25(OH)D, even after excluding children <6 months. These findings indicate that vitamin D status among children with SAM is significantly influenced by age, seasonality, cerebral palsy, and geographical location. To facilitate management and improve clinical outcomes in these children, we recommend further investigation into whether the current vitamin D content in ready-to-use therapeutic foods is sufficient for all children and whether additional supplementation improves clinical outcomes, especially in high-risk groups. If deficiency is present, we recommend that children be managed in accordance with local guidelines and policies.

## Introduction

Severe Acute Malnutrition (SAM) encompasses two forms of acute malnutrition: severe wasting and nutritional oedema [1]. According to World Health Organisation (WHO) growth standards, SAM in children aged 6–59 months is defined as a mid-upper arm circumference (MUAC) below 115 mm or a weight-for-height z-score (WHZ) below −3 [2], with or without bilateral oedema. Children with SAM who have severe oedema, poor appetite, and/or medical complications such as diarrhoea, pneumonia or metabolic problems need urgent medical intervention and hospitalisation for management [1]. According to the WHO, an estimated 19 million children below the age of 5 years are living with SAM, and 400,000 die each year [3]. A review by Asebe *et al.* reported that 2.1% (95% CI: 2.0, 2.2%) of children aged between 6 and 59 months in sub-Saharan Africa are affected by severe wasting [4], while in Zambia, the Demographic and Health Survey (DHS) of 2024 reported that approximately 1% of Zambian children are living with SAM [5]. In Zimbabwe, approximately 15,000 children are treated for severe wasting annually [6].

SAM is accompanied by micronutrient deficiencies, including zinc, selenium, iron [7–9] and Vitamin D [10]. In children with SAM, Vitamin D helps maintain calcium and phosphorus balance by promoting their absorption in the intestines and kidneys [11]; while deficiency complicates recovery [10]. Children with SAM have an altered immune response to bacterial antigens [12], which increases their risk of mortality. Given that Vitamin D exerts anti-inflammatory and antimicrobial effects [13–17], understanding vitamin D status in children recovering from SAM may be clinically relevant in this high-risk group, as it may contribute to recovery. Besides clinical management, treatment of SAM includes nutritional supplementation via Ready-to-use therapeutic food (RUTF), which contains 15–22ug/100g of Vitamin D (Cholecalciferol) [18]. Whether this is sufficient for all children with SAM is unclear. In low- and middle-income countries (LMICs), responsiveness to RUTF may be impeded by environmental enteropathy, a condition characterised by increased malabsorption [19] and common among people living in unsanitary environments [20–22].

Vitamin D status is evaluated using serum 25‑hydroxyvitamin D (25(OH)D), the most abundant and stable circulating metabolite, with a half-life of approximately three weeks [23,24]. Using a cut-off of <20ng/mL (where nmol/L = ng/mL*2.5), a study in Tunisia found that 84 (97%) of the women and 85 (98%) of the neonates were vitamin D deficient, with 76 (87%) and 78 (90%) being severely deficient (<12ng/mL) [25]. Although the prevalence of vitamin D deficiency (<20 ng/mL) declined from 76.4% at 6 weeks of age, infants who were exclusively breastfed at 6 weeks of age had a higher risk of vitamin D deficiency than formula-fed infants [26]. In Nigerian mothers and their infants, the duration of exclusive breastfeeding was inversely associated with plasma vitamin D levels [27]. These findings not only provide evidence of high levels of vitamin D deficiency in some parts of Africa but also highlight a higher risk of vitamin D deficiency in exclusively breastfed infants as a high-risk group.

Circulating 25(OH)D levels vary with sunlight exposure and can exhibit seasonal patterns [28–30]. In Africa, these variations can be attributed to heterogeneous climates, diets, latitudes, geographies, and skin pigmentation [31]. Vitamin D deficiency and thresholds depend on several factors, including the population of interest and condition [32–34], which complicates comparisons across settings. However, evidence of vitamin D supplementation or deficiency in Zambian or Zimbabwean children is limited.

Although children with SAM are discharged from hospital only after clinical recovery and stabilisation [3,8], post-discharge mortality persists [35], potentially reflecting delayed immune restoration relative to nutritional recovery [12]. Children with SAM may also experience impaired neurodevelopment [36]; however, despite the known roles of vitamin D in immune function and neurodevelopment, vitamin D status at discharge remains poorly described. We aimed to i) determine the prevalence of 25(OH)D deficiency at discharge and ii) identify determinants associated with vitamin D concentrations in Zambian and Zimbabwean children recovering from SAM, thereby addressing an important gap in post-discharge care.

## Materials and methods

### Study design

This was a cross-sectional study nested in the Health Outcomes, Pathogenesis and Epidemiology of Severe Acute Malnutrition (HOPE-SAM) study, which has been described in detail elsewhere (https://osf.io/29uaw/.) [37,38]. Briefly, HOPE-SAM was a longitudinal prospective cohort study of 745 children under five years of age hospitalised for complicated SAM at the University Teaching Hospital in Zambia and Parirenyatwa General and Harare Children’s Hospitals in Zimbabwe, recruited between 14th July 2016 and 15th March 2018 [35].

The HOPE-SAM study enrolled 755 children with SAM across both countries. Of these, three were ineligible, two were co-enrolled in other studies, three exited before baseline and two died, leaving 745 children (242 in Zambia and 503 in Zimbabwe) who were included in the main study [35,37]. However, of the 745 children, 70 died, and 26 exited from the study while in hospital, leaving 649 children from both countries who were discharged alive (S1 Fig). Of the 649 children at discharge, only 442 had plasma samples available for this cross-sectional analysis of 25(OH)D in both Zambia (n = 101) and Zimbabwe (n = 341) (S1 Fig).

### Inclusion and exclusion criteria

This sub-study included children at hospital discharge who had recovered clinically and were to be managed as outpatients after meeting the World Health Organisation (WHO) criteria of WHZ > –2 and/or MUAC >125mm without oedema for at least two weeks [39]. As screening was conducted in the main study, children in this cross-sectional analysis were excluded only if they had insufficient plasma samples for the 25(OH)D analysis.

### Laboratory analysis

Plasma concentrations of 25(OH)D were quantified in singlicate using ELISA kits from the Immunodiagnostic System (reference number AC-575F1; Lot number J45789) according to the manufacturer’s instructions. Two assay controls provided with every kit were included and analysed with each plate, yielding comparable results between the two sites. Each plate was analysed using the same settings on Biotek ELx808 readers located at Tropical Gastroenterology and Nutrition Group (TROPGAN) laboratories and the Zvitambo Institute for Maternal and Child Health Research laboratory in Zambia and Zimbabwe, respectively. Sample concentrations were determined using a non-linear 4-parametric logistic regression (4-PL) standard curve.

### Statistical analysis

Participant clinical and demographic characteristics were collected using a questionnaire that was completed by the caregiver and administered by the study nurse at baseline. Data were presented as medians with interquartile ranges (IQRs) or as proportions with 95% confidence intervals (CIs). For proportions, statistical significance was assessed using Pearson’s Chi-square test. As 25(OH)D concentrations were not normally distributed, the Mann-Whitney or Kruskal-Wallis tests were used to compare medians between and across groups, respectively, with P < 0.05 considered significant. Values of 25(OH)D ≥ 50 nmol/L were classified as sufficient [40,41], while concentrations < 50 nmol/L were considered deficient. Seasonality at discharge was assessed by categorising months into three seasons. In Zimbabwe [42], the rainy season is from November to April, the cool season from May to August, and the hot season from September to October. In Zambia [43], the rainy season is from December to April, the cool season from May to August, and the hot season from September to November. Seasons in both countries extensively overlap, with slightly longer cool and hot seasons in Zimbabwe and Zambia, respectively.

Despite the Shapiro-Wilk test indicating that the data were not normally distributed, a visual inspection of the 25(OH)D concentrations using a histogram and Q–Q plot revealed no substantial deviations from normality. Log transformation improved the distribution of the residuals, with Q–Q plots showing approximate normality, with minor deviations remaining at the tails. Variance inflation factors (VIFs) were examined to assess multicollinearity, and all VIFs were below 3 (S1 Table). Multivariable regression models were specified a priori based on known determinants (country, HIV infection, age, sex, oedema status, cerebral palsy and seasonality), with log-transformed 25(OH)D concentrations as the dependent variable. As children <6 months were more likely to have been exclusively breastfed, a sensitivity analysis excluding children <6 months was undertaken. All beta coefficients and 95% CIs are reported as adjusted (adj) ratios. All statistical analyses were performed using Stata 17.0 (Stata Corp, College Station, TX), and all graphs were created using GraphPad Prism 10.

### Ethical considerations

Ethical approval for the study was obtained from the University of Zambia Biomedical Research Ethics Committee (010-02-16) and the Medical Research Council of Zimbabwe. The ethics committee of Queen Mary University of London, U.K., also provided a non-binding advisory review. Before any child could be enrolled in this study, written informed consent was obtained from the caregivers following careful explanation by trained study nurses using standardised consent forms reviewed and approved by our ethics committees. Comprehension of the consent form was checked using a checklist before consent forms were signed.

## Results

### Characteristics of the study participants

Among 442 children recruited in Zambia and Zimbabwe, we found that age, sex and clinical phenotype did not differ significantly (Table 1). Zambia had significantly more children who were living with HIV than Zimbabwe (27 (26.7%) and 52 (15.2%), respectively; P = 0.008).

**Table 1:** Table showing characteristics for children who were analysed at discharge.

	Zambia	Zimbabwe	*P*
*Number of children* who had their plasma 25(OH)D analysed at discharge	**n=101**	**n=341**	
*Age (months)*	17. 3 [14.1 –24.0]	18.7 [13.4–23.4]	0.899
*Sex (M: F)*	59:42	174:167	0.191
*WHZ,* Median [IQR]	−2.1 [−2.8, −1.3]	−2.2 [−3.1, −1.2]	0.397
*MUAC (mm),* Median [IQR]	122.3 [112.7–131.0]	123.0 [114.0–134.3]	0.387
*Oedema present, n (%)*	8 (7.9 %)	28 (8.2 %)	0.919
*Cerebral Palsy, n (%)*	5 (5.0 %)	22 (6.5 %)	0.580
*Primary Caregiver*			0.073
Mother	98 (97.0%)	310 (90.9%)	
Father	1 (1.0%)	2 (0.6%)	
Other	2 (2.0%)	29 (8.5%)	
*HIV status, n (%)*			**0.008**
Positive	27 (26.7 %)	52 (15.2 %)	
Negative	74 (73.3 %)	289 (84.8 %)	
*Season of Sampling*			0.363
Cool	13 (12.9 %)	60 (17.6 %)	
Hot	24 (23.8 %)	64 (18.5 %)	
Rainy	64 (63.4 %)	216 (63.8 %)	

Categorical variable analysis using Pearson’s Chi-squared test and Mann-Whitney for continuous variables. WHZ: Weight for height Z score; MUAC: Mid-upper-arm circumference; IQR: Interquartile range.

Sex, Cerebral Palsy, Primary caregiver, and HIV status; Zimbabwe: n = 341 and Zambia: n = 101; Age, WHZ, MUAC, Oedema and season of sampling: Zimbabwe: n = 340 and Zambia: n = 101.

P values < 0.05 considered significant.

### 25(OH)D concentrations in Zambia and Zimbabwe

**Fig 1:**
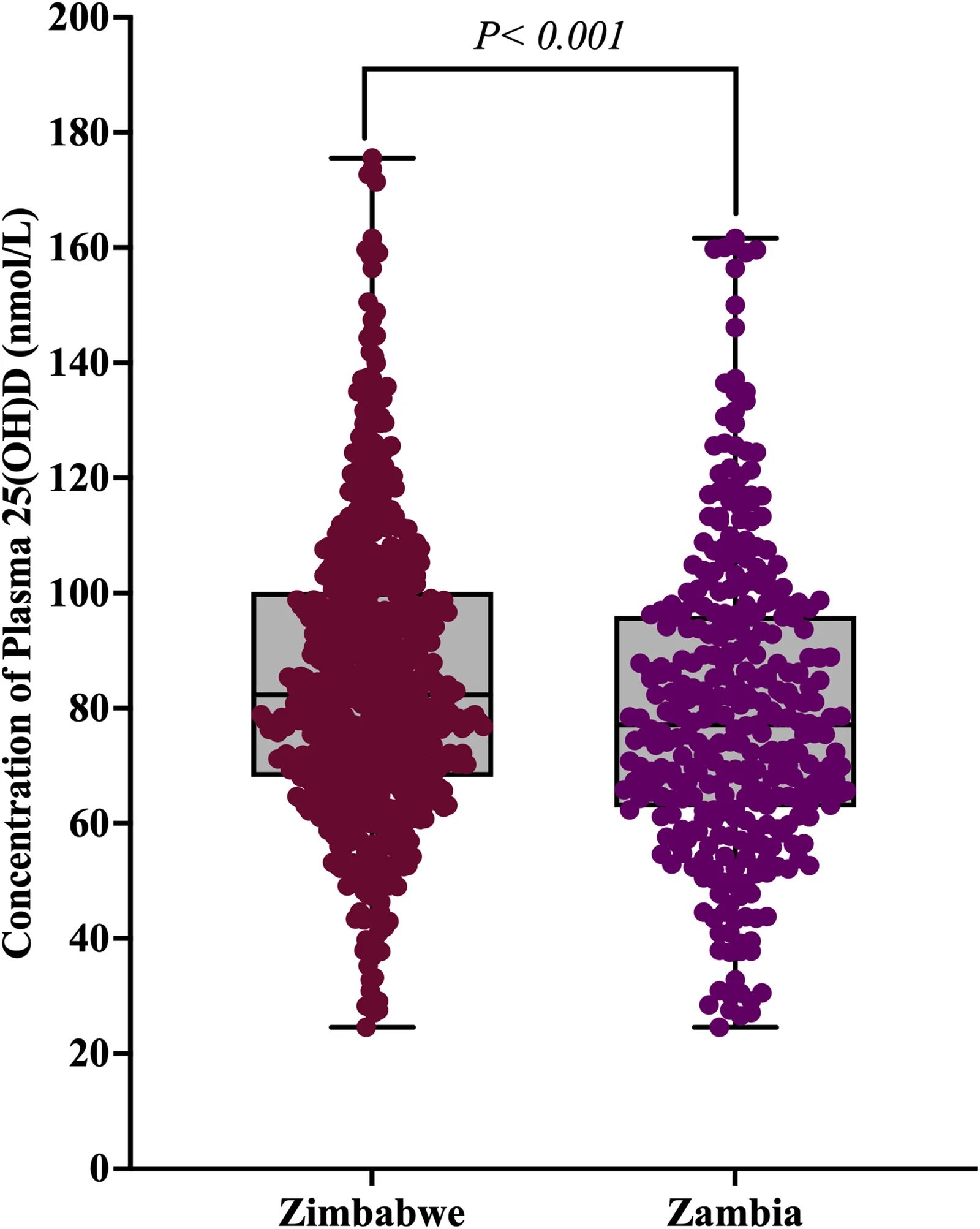
Plasma concentrations of 25(OH)D at discharge in Zimbabwean and Zambian Children. The Mann-Whitney test was used; Zambian (n = 101) and Zimbabwean (n = 341) children were included.

Concentrations of 25(OH)D were higher in the Zambian (89.1 nmol/L; IQR 72.3, 107.7) than in Zimbabwean children (77.1 nmol/L; IQR 63.1, 95.9) by 12.0 nmol/L (95% CI 5.1, 18.9; P = 0.001; Fig 1). Between the two Zimbabwean hospitals, Parirenyatwa (78.9 nmol/L; IQR 62.3, 97.6) and Harare Children’s (75.8 nmol/L; IQR 63.7, 92.3) hospitals, plasma concentrations of 25(OH)D did not differ significantly. However, 25(OH)D concentrations from these hospitals were significantly lower than those from UTH (89.1 nmol/L; IQR 75.3, 107.7) (S2 Fig). Of the 442 children, we found concentrations consistent with deficiency in 33 (7.5%; 95% CI 5.2, 10.3), all of whom were from Zimbabwe. Hence, excluding all Zambian children, 33/341 (9.7%; 95% CI 6.8–13.3) of Zimbabwean children were deficient.

### Effect of seasonality on circulating 25(OH)D concentrations

**Fig 2:**
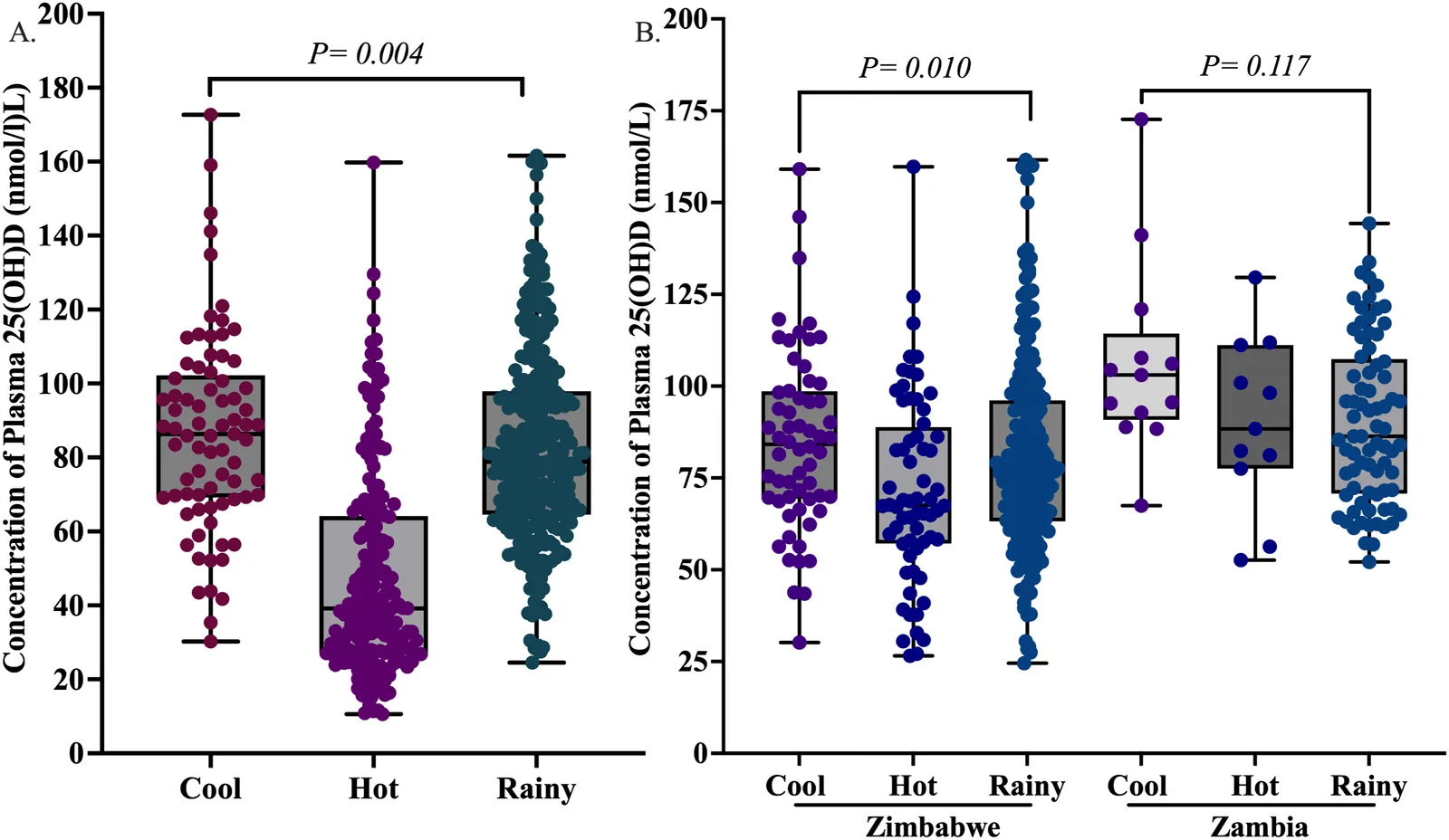
Plasma concentrations of 25(OH)D at discharge by season. a. A combined comparison in both Zambian and Zimbabwean children according to the season when the samples were collected: cool (n = 73), hot (n = 88) and rainy (n = 280): Kruskal-Wallis was used. b. A separate comparison of 25(OH)D concentrations according to the season of sample collection in each country. Cool; Zambia (n = 13) vs Zimbabwe (n = 60); Hot; Zambia (n = 24) vs Zimbabwe (n = 64); Rainy; Zambia (n = 64) vs Zimbabwe (n = 216). Kruskal-Wallis was used.

In Zambia and Zimbabwe combined, plasma 25(OH)D concentrations were significantly lower (P = 0.004) in the hot (69.3 nmol/L; IQR: 57.56–96.17) season than in the rainy (79.1 nmol/L; IQR: 65.4–98.0) or cool (88.4 nmol/L; IQR: 69.9–103.0) seasons (shown in Fig 2a). This seasonal effect was country-specific: Zimbabwe had significantly higher concentrations in the Cool season (84.2 nmol/L, IQR: 69.5–98.5; versus Hot season: 67.5 nmol/L, IQR: 57.3–88.0; versus Rainy season; 77.8 nmol/L; IQR: 63.3–96.0; P = 0.010) but the concentrations did not differ significantly among Zambian children (Cool: 103.0 nmol/L; IQR: 92.8–107.7; versus Hot: 88.4 nmol/L; IQR: 77.5–111.2; versus Rainy; 86.4 nmol/L; IQR: 71.0–106.7; P = 0.117) (shown in Fig 2b).

### Effect of clinical factors on circulating 25(OH)D concentrations

**Fig 3:**
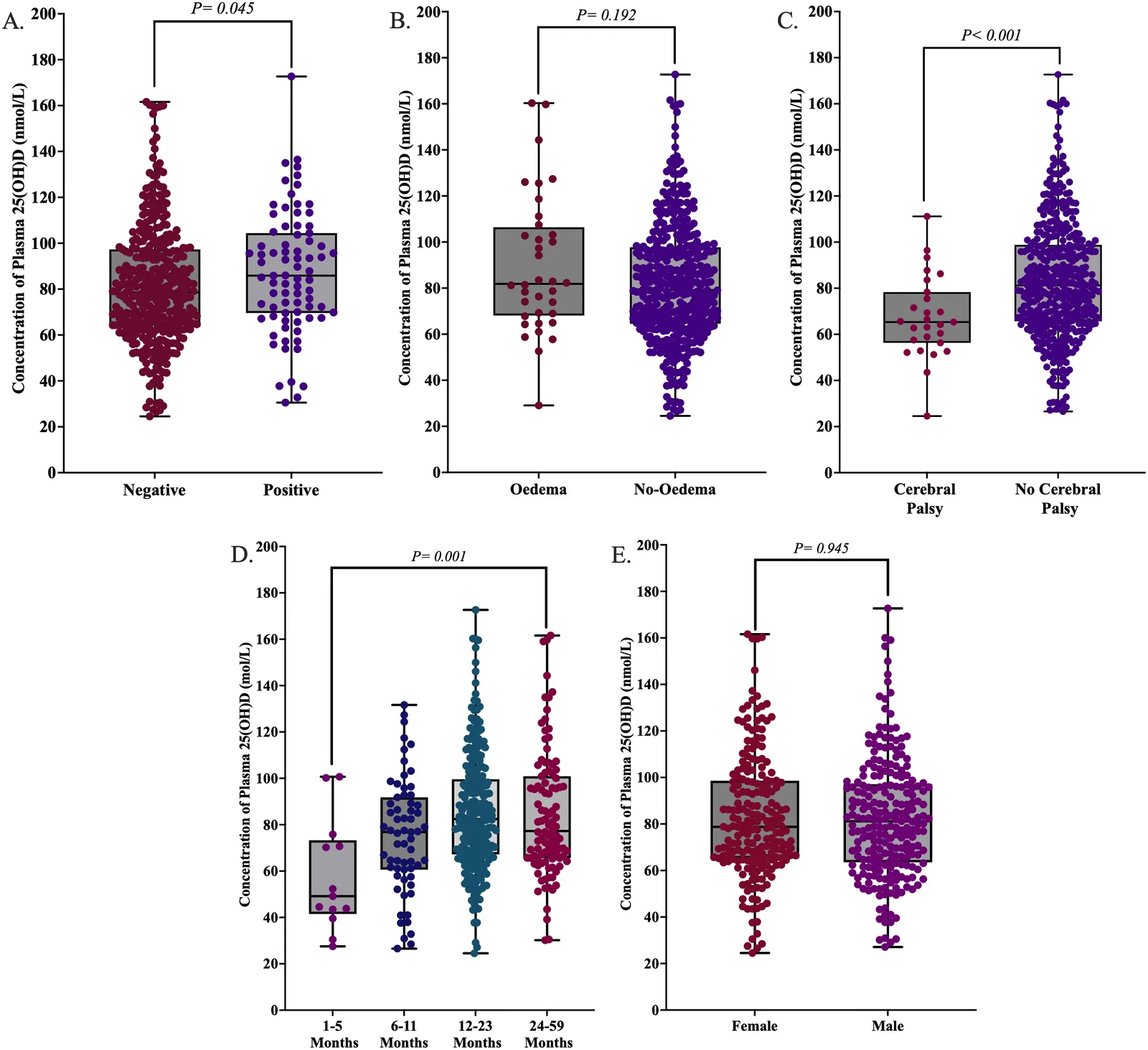
Plasma concentrations of 25(OH)D at discharge according to HIV status, oedema status, cerebral palsy status, age, and sex. a). HIV+ (n = 79) and HIV- (n = 363); b). Oedema (n = 36) and no-Oedema (n = 405); c) Cerebral palsy (n = 27) and No Cerebral palsy (n = 415); d). Age: 1-5 months (n = 13), 6-11months (n = 63), 12-23months (n = 262), and 24-59 months (n = 103); Male (n = 234) and Female (n = 208).

We found no evidence of a difference in 25(OH)D concentrations between male (81.2, IQR: 63.7–96.9) and female children (78.8, IQR: 65.3–98.5; 0.945: Fig 3e). However, 25(OH)D concentrations were significantly lower in children aged 1–5 months (49.1 nmol/L, IQR 43.5–70.7), 6–11 months (70.5 nmol/L, IQR 56.3–89.3 nmol/L) and 24–59 months (73.3 nmol/L, IQR 63.9–9.6 nmol/L) relative to those aged 12–23 months (79.8 nmol/L, IQR 66.2–96.8 nmol/L; P = 0.04; Fig 3d). 25(OH)D concentrations were higher in children with HIV (85.9 nmol/L, IQR: 69.6–104.4) than in children without HIV (78.6 nmol/L, IQR: 64.2–97.3; P = 0.05; Fig 3a). Children with cerebral palsy had lower (65.3 nmol/L, IQR 56.3–78.3) circulating 25(OH)D concentrations than children who did not have cerebral palsy (81.2 nmol/L, IQR 65.7–98.8; P < 0.001; Fig 3c). Circulating plasma concentrations of 25(OH)D did not differ between children admitted with oedematous (81.9 nmol/L; IQR: 68.4–105.3) versus non-oedematous SAM (79.1 nmol/L; IQR: 64.6, 97.0; *P* = 0.17; Fig 3b).

### Mortality

To determine if there was an association between vitamin D and mortality, plasma 25(OH)D concentrations at discharge were compared between the 36 children who were known to have died over the subsequent period of follow-up and the 406 children who were known to have survived. Plasma 25(OH)D concentrations were similar (P = 0.905) in those who survived (79.4 nmol/L, IQR: 64.9–98.0) and those who died (80.95 nmol/L, IQR: 62.6–102.8).

### Multivariable linear regression analysis

To assess the relative importance of country, HIV infection, oedema status, age, sex, cerebral palsy, and seasonality, these variables were included in a regression model, with log-transformed 25(OH)D concentrations as the dependent variable. In this model, residence in Zambia (adj ratio; 1.2, 95%CI 1.1, 1.2; P < 0.001), being older than 6 months (adj ratio; 6–11 months: 1.3, 95% CI 1.1, 1.6; P = 0.003, 12–23 months: 1.5, 95% CI 1.3, 1.8; P < 0.001 and 24–59 months: 1.5, 95% CI 1.3, 1.8; P < 0.001), absence of cerebral palsy (adj ratio; 1.2, 95%CI 1.1, 1.4; P = 0.002) and sampling during the cool season (adj ratio; 1.2; 95% CI 1.1, 1.3; P = 0.002) were associated with significantly higher 25(OH)D concentrations (S2 Table). Using the same model, excluding children <6 months, residence in Zambia (adj ratio; 1.2, 95% CI 1.0, 1.2; P < 0.001), older than 11 months (adj ratio; 12–23months: 1.1, 95% CI 1.0, 1.2; P = 0.004 and 24–59 months: 1.1, 95% CI 1.0, 1.2; P = 0.015), absence of cerebral palsy (adj ratio; 1.2, 95% CI 1.1, 1.4; P = 0.002) and sampling during the cool season (adj ratio; 1.2; 95% CI 1.1, 1.3; P = 0.002) were associated with higher plasma concentrations of 25(OH)D (S3 Table).

## Discussion

Vitamin D has anti-inflammatory and immunomodulatory properties [44] and, therefore, may have implications for clinical recovery. We set out to determine if deficiency is a feature of SAM in two countries in southern Africa. Among 442 children drawn from the HOPE-SAM study, at hospital discharge, 7.5% had vitamin D deficiency (<50 nmol/L), but none were from Zambia. Sampling during the cool season and older age were associated with higher circulating 25(OH)D concentrations, whilst cerebral palsy was associated with lower concentrations.

The prevalence of vitamin D deficiency we report here is lower than that reported in previous studies of malnourished and well-nourished school-going children elsewhere in Africa [25]. A study conducted on 117 malnourished and 41 non-malnourished children aged 6–24 months in Uganda reported comparable vitamin D levels between the two groups (32.5 nmol/L (±12.0 SD) and 32.2 nmol/L (10.9 SD); P = 0.868) [45]. In 828 hospitalised malnourished adults in Switzerland, the prevalence of vitamin D deficiency (<50 nmol/L) was 58.2%, and these patients were more likely to die before 180 days (odds ratio (OR) 1.42; 95% CI 1.03–1.94, *P* = 0.03) than adults with sufficient vitamin D levels [46]. These studies highlight the importance of Vitamin D in health and survival, suggesting that supplementation may enhance clinical outcomes.

In two separate studies involving older Mongolian school-going children, increases in Vitamin D levels after supplementation were observed, but with no significant differences between the Vitamin D-supplemented and placebo groups in terms of improvement in growth, body composition, or pubertal development [47], nor in height-for-age z-score or body mass index-for-age z-score in South African school-going children aged 6–11 years [48]. Nonetheless, vitamin D supplementation was associated with improved wasting, evidenced by significant weight gain in children with SAM [49]. Also, a clinical trial from Pakistan suggests that adjunctive vitamin D supplementation may improve outcomes in children receiving standard therapy for uncomplicated SAM and may be essential for growth recovery and neurodevelopment [50].

Currently, the prevalence of cerebral palsy is estimated to be around 3.4% in LMIC [51]. Hence, children who have both SAM and cerebral palsy, as observed in this and other studies [45,52–57], often have lower circulating 25(OH)D concentrations and may benefit from vitamin D supplementation. However, both conditions are complex, and clinical presentation and requirements may vary from one child to another. Children with cerebral palsy may require higher levels of vitamin D supplementation than those presenting with SAM only, as they already have pre-existing reduced bone mineral density, bone fragility, osteopenia, and rickets [56]. Hence, further investigations to determine optimal supplementation doses are warranted.

Possible reasons for the lower 25(OH)D concentrations in Zimbabwean children compared with Zambian children may include lower vitamin D intake, greater malabsorption, and/or inadequate sunlight exposure, as well as variations in cultural and socio-economic factors [58]. These factors, in turn, may include diet [59], dress, health-seeking and other behaviours. Additionally, all children <6 months old (n = 13) were from Zimbabwe, which may in part explain the differences. Although caregivers influence the feeding behaviour of children [60] and maternal practices being linked to infants’ health outcomes [61], in this study, primary caregiver type differed modestly between countries, with no evidence that caregiver type explained vitamin D deficiency; with only one child not cared for by a biological parent being vitamin D deficient. Lower vitamin D levels have previously been associated with colder seasons due to reduced sunlight exposure [62]. However, we report lower 25(OH)D concentrations during the hot season than the cool and rainy seasons, which may be explained by several factors, including behavioural avoidance of sun exposure, which may reduce cutaneous vitamin D synthesis despite higher ambient UV availability [63,64]. Although the differences may be minimal, Lusaka lies at 15.4°S and Harare at 17.8°S [65,66]. Furthermore, since 1930, annual temperatures in Harare have been 1–4°C cooler than in Lusaka, with higher precipitation in Zimbabwe than in Zambia (S1–S4 Tables) [67]. This may be explained by the altitude differences between Harare (1,483 m) and Lusaka (1,280 m). Though diets in Zambia and Zimbabwe are very similar [68], as are therapeutic formulations for treatment of SAM, it is also possible that the vitamin D content of agricultural products in the two countries vary seasonally [69], and the differences between countries could be multifactorial.

In this study, the youngest group (1–5 months) had the lowest circulating 25(OH)D concentrations, which may be attributed to exclusive breastfeeding and limited sunlight exposure due to age. A study in Tunisia among women and their neonates reported mean 25(OH)D serum levels of 6.82 ± 5.14 ng/mL (range 3.60–23.77) and 5.92 ± 4.15 ng/mL (range 3.60–22.28) (P = 0.001), respectively [25]. In Tanzanian children born to HIV-negative women, children <6 months who were exclusively breastfed had double the risk of Vitamin D deficiency compared to children who received formula, and younger children were more likely to have lower 25(OH)D concentrations [26]. Hence, re-evaluating whether the current levels of vitamin D in RUTF are sufficient for all children is essential, as younger children may need more than they receive currently, in which case RUTF reformulation or supplementation could be considered.

In this study, 25(OH)D plasma concentrations did not differ significantly between children who were living with HIV and children without HIV. However, 51/78 of the children living with HIV were taking antiretroviral therapy (ART). In contrast to our findings, previous studies have reported a higher prevalence of vitamin D deficiency among people living with HIV, with consequences including reduced bone mineral density, osteoporosis, and osteopenia [70–73]. We found no evidence of sex-related differences in vitamin D status; current evidence is inconsistent, with some studies reporting higher vitamin D levels in males than in females, and others reporting higher levels in females than in males [74–76]. Additionally, although we found no significant difference in 25(OH)D concentrations between oedematous and non-oedematous SAM, oedematous SAM is associated with poorer inpatient clinical outcomes [77]. A previous study reported that children with marasmus (non-oedematous SAM) were 10.8 times (OR 10.8, CI = 1.356–86.236, *p* = 0.03) more likely to have Vitamin D deficiency than those with kwashiorkor or marasmic-kwashiorkor [78], but our study did not find such a difference. Lastly, although we found no significant differences in 25(OH)D levels between children who survived or died during recovery, low plasma concentrations of 25(OH)D are inversely associated with all-cause mortality [79]. Although different from SAM, a study in children admitted to the paediatric intensive care unit reported higher vitamin D levels in survivors than in non-survivors [80]. Also, in children who were on dialysis, 64.7% of those who were vitamin D deficient died, whereas all those with sufficient levels survived [81]. Further investigations on the associations between HIV, sex, oedema status, mortality and vitamin D in malnourished children are needed.

There are significant limitations to this study. We did not have the opportunity to include a well-nourished community or hospital control group (admitted for reasons other than SAM). We also acknowledge that differences in sample sizes between Zambia and Zimbabwe reduce the power to draw firm conclusions across countries. This is especially true of cerebral palsy and oedema, as the numbers of affected children were small. Further investigation is needed to determine whether these subgroup differences persist. Lastly, although all experiments included assay controls and were analysed using the same ELISA reader with the same instrument settings, it is possible that running our samples singly and processing samples in two different laboratories may contribute to the difference in concentrations between Zambia and Zimbabwe.

## Conclusion and recommendations

Vitamin D deficiency (<50 nmol/L) was present in 7.5% of children recovering from SAM at hospital discharge. We found no evidence that 25(OH)D concentrations differed by sex, HIV status, oedema status, or subsequent survival. In contrast, 25(OH)D concentrations were associated with age, country, season, and cerebral palsy. These findings suggest that vitamin D status at discharge varies across subgroups and may warrant targeted attention during recovery. Future studies should evaluate whether the current vitamin D content in ready‑to‑use therapeutic foods is sufficient for all children and whether additional supplementation improves clinical outcomes, especially in higher‑risk groups. Where deficiency is identified, children should be monitored and managed in accordance with local clinical guidelines and policies. Additionally, seasonality affects vitamin D levels, but it is unclear if vitamin D deficiency resolves with time or change in season. Longitudinal studies would clarify seasonal variation over time and its implications for post‑discharge care. Lastly, although no association was observed between vitamin D status and mortality, children identified as deficient at hospitalisation should still be closely monitored.

## Supporting information

S1 Fig Study schema. Summary of the number of children living with SAM included in the 25(OH)D analysis in both Zambia and Zimbabwe at hospital discharge (Zambia: n = 101 and Zimbabwe: n = 341).
(PDF)

S2 Fig Plasma 25(OH)D concentrations at discharge by hospital of admission. (PDF)

S1 Table Variance inflation factor summary. (PDF)

S2 Table Multivariable linear regression analysis, including all children. (PDF)

S3 Table Multivariable linear regression analysis excluding children aged <6 months. (PDF)

S4 Table Mean seasonal temperatures recorded in Lusaka and Harare, 1901–2020. (PDF)

S5 Table Minimum seasonal temperatures in Lusaka and Harare, 1901–2020. (PDF)

S6 Table Maximum seasonal temperatures in Lusaka and Harare, 1901–2020. (PDF)

S7 Table Seasonal precipitation in Lusaka and Harare, 1901–2020. (PDF)

S8 Table Dataset used for all analyses presented in this manuscript. (XLSX)
