# Supplementary material for: A cross‑sectional analysis of vitamin D status and associated factors in children recovering from severe acute malnutrition in Zambia and Zimbabwe
Source: PLoS One. 2026 Aug 28;21(8):e0355404. doi: 10.1371/journal.pone.0355404 (PMC13524285; doi:10.1371/journal.pone.0355404)
Supplement: Supplementary file 1 [file pone.0355404.s001.pdf]

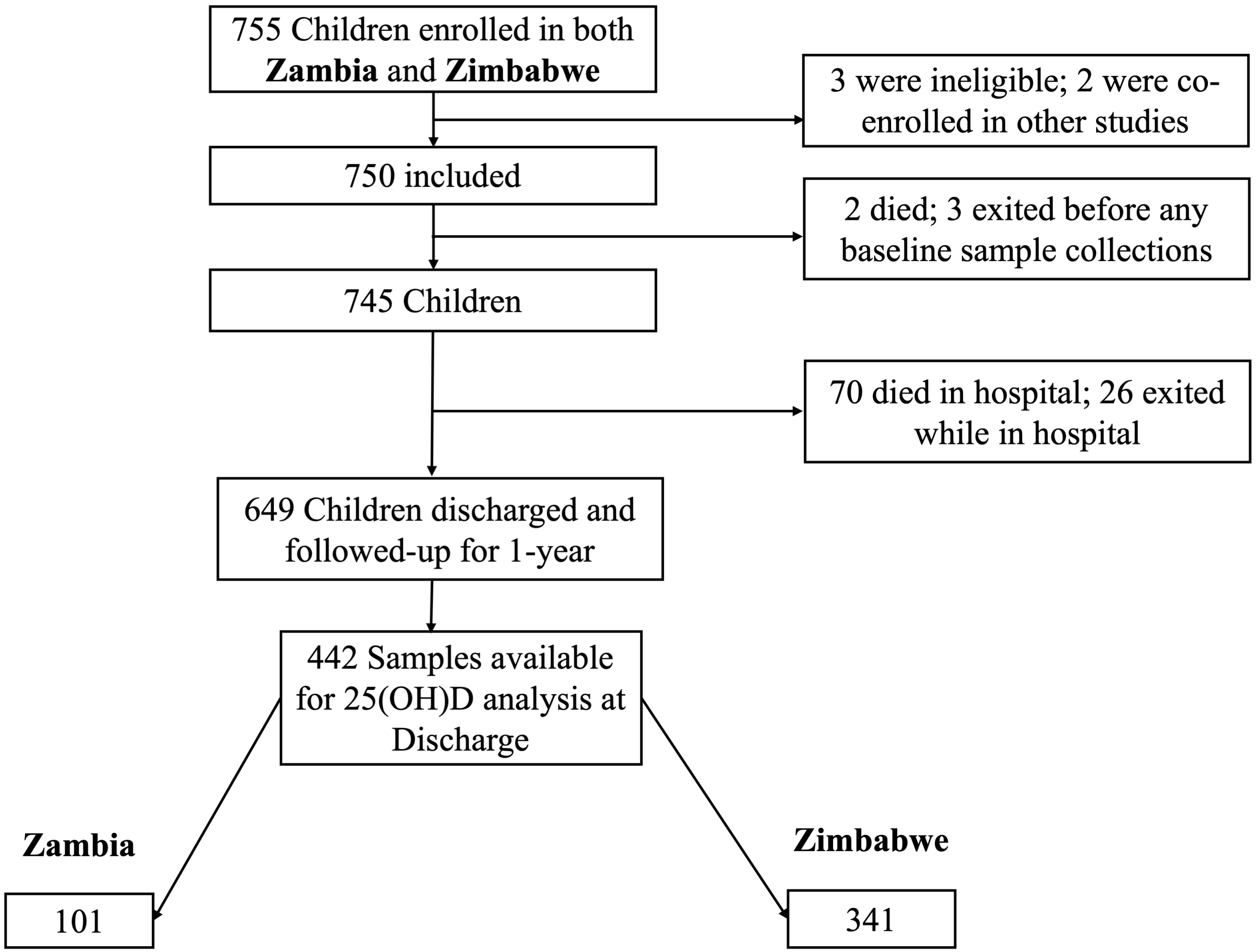

S1 Fig. Study schema: Summary of the number of children living with SAM included in the 25(OH)D analysis in both Zambia and Zimbabwe at hospital discharge (Zambia: n=101 and Zimbabwe: n=341).
