# Supplementary material for: A cross‑sectional analysis of vitamin D status and associated factors in children recovering from severe acute malnutrition in Zambia and Zimbabwe
Source: PLoS One. 2026 Aug 28;21(8):e0355404. doi: 10.1371/journal.pone.0355404 (PMC13524285; doi:10.1371/journal.pone.0355404)
Supplement: Supplementary file 3 [file pone.0355404.s003.pdf]

S1 Table. Variance inflation factor summary

Variable	*VIF*	*1/VIF*
HIV Status	1.06	0.9
Cerebral Palsy	1.05	1.0
Age	1.04	1.0
Country of residence	1.03	1.0
Season	1.02	1.0
Oedema Status	1.01	1.0
Gender	1.01	1.0
Mean VIF	*1.03*	
