# Supplementary material for: A cross‑sectional analysis of vitamin D status and associated factors in children recovering from severe acute malnutrition in Zambia and Zimbabwe
Source: PLoS One. 2026 Aug 28;21(8):e0355404. doi: 10.1371/journal.pone.0355404 (PMC13524285; doi:10.1371/journal.pone.0355404)
Supplement: Supplementary file 4 [file pone.0355404.s004.pdf]

**S2 Table. Multivariable linear regression analysis, including all children**

| | *N= 441* | *Coefficient* | *Standard error* | *t* | *95% CI* | *P Value* |
|---|---|---|---|---|---|---|
| *Sex* | Male | | | *Ref* | | |
| | Female | 1.0 | 0.0 | 0.4 | 1.0, 1.1 | 0.699 |
| *Country* | Zimbabwe | | | *Ref* | | |
| | Zambia | 1.2 | 0.0 | 4.2 | 1.1, 1.2 | **< 0.001** |
| *Age* | 1-5 Months | | | *Ref* | | |
| | 6-11 Months | 1.3 | 0.1 | 3.0 | 1.1, 1.6 | **0.003** |
| | 12-23 Months | 1.5 | 0.1 | 4.6 | 1.3, 1.8 | **< 0.001** |
| | 24-59 Months | 1.5 | 0.1 | 4.4 | 1.3, 1.8 | **< 0.001** |
| *HIV Status* | Negative | | | *Ref* | | |
| | Positive | 1.0 | 0.0 | 0.9 | 1.0 , 1.1 | 0.350 |
| *Cerebral Palsy* | With Cerebral Palsy | | | *Ref* | | |
| | Without Cerebral Palsy | 1.2 | 0.1 | 3.0 | 1.1, 1.4 | **0.002** |
| *Oedema* | No Oedema | | | *Ref* | | |
| | With Oedema | 1.1 | 0.1 | 1.3 | 1.0, 1.2 | 0.185 |
| *Season* | Hot | | | *Ref* | | |
| | Cool | 1.2 | 0.1 | 3.1 | 1.1, 1.3 | **0.002** |
| | Rainy | 1.1 | 0.0 | 1.7 | 1.0, 1.2 | 0.097 |

Adjusted R-squared Value= 0.1,  R-squared Value= 0.1 and Prob > F =  <0.001
Multivariable regression was done with '*Ref*' as the reference group
P values < 0.05 was considered significant
