# Supplementary material for: A cross‑sectional analysis of vitamin D status and associated factors in children recovering from severe acute malnutrition in Zambia and Zimbabwe
Source: PLoS One. 2026 Aug 28;21(8):e0355404. doi: 10.1371/journal.pone.0355404 (PMC13524285; doi:10.1371/journal.pone.0355404)
Supplement: Supplementary file 5 [file pone.0355404.s005.pdf]

S3 Table. Multivariable linear regression analysis excluding children aged <6 months

	N= 428	*Coefficient*	*Standard error*	*t*	*95% CI*	*P Value*
Sex	Male			*Ref*		
	Female	1.0	0.0	0.6	1.0, 1.1	0.540
Country	Zimbabwe			*Ref*		
	Zambia	1.2	0.0	4.2	1.1, 1.2	**< 0.001**
Age	6-11 Months			*Ref*		
	11-23 Months	1.1	0.0	2.9	1.0, 1.2	**0.004**
	24-59 Months	1.1	0.1	2.4	1.0, 1.2	**0.015**
HIV Status	Negative			*Ref*		
	Positive	1.0	0.0	0.9	1.0, 1.1	0.369
Cerebral Palsy	With Cerebral Palsy			*Ref*		
	Without Cerebral Palsy	1.2	0.1	3.1	1.1, 1.4	**0.002**
Oedema	No Oedema			*Ref*		
	With Oedema	1.1	0.1	1.3	1.0, 1.2	0.185
Season	Hot			*Ref*		
	Cool	1.2	0.1	3.1	1.1, 1.3	**0.002**
	Rainy	1.1	0.0	1.7	1.0, 1.2	0.096

Adjusted R-squared Value= 0.1, R-squared Value= 0.1 and Prob > F = <0.001
Multivariable regression was done with '*Ref*' as the reference group
P values < 0.05 was considered significant
