# Supplementary material for: A cross‑sectional analysis of vitamin D status and associated factors in children recovering from severe acute malnutrition in Zambia and Zimbabwe
Source: PLoS One. 2026 Aug 28;21(8):e0355404. doi: 10.1371/journal.pone.0355404 (PMC13524285; doi:10.1371/journal.pone.0355404)
Supplement: Supplementary file 6 [file pone.0355404.s006.pdf]

**S4 Table. Mean seasonal temperatures recorded in Lusaka and Harare, 1901–2020**

| Observed Average Seasonal Mean Temperature | | | | | | | | | | | | | | | | |
|---|---|---|---|---|---|---|---|---|---|---|---|---|---|---|---|---|
| | **1991-2020** | | | | **1961-1990** | | | | **1931-1960** | | | | **1901-1930** | | | |
| **Units: °C** | **DJF** | **MAM** | **JJA** | **SON** | **DJF** | **MAM** | **JJA** | **SON** | **DJF** | **MAM** | **JJA** | **SON** | **DJF** | **MAM** | **JJA** | **SON** |
| Lusaka, Zambia | 25.02 | 22.87 | 19.46 | 26.36 | 24.27 | 22.2 | 18.59 | 25.49 | 24.41 | 22.36 | 18.51 | 25.52 | 24.53 | 22.2 | 18.48 | 25.6 |
| Harare, Zimbabwe | 22.01 | 19.35 | 15.37 | 21.54 | 21.28 | 18.65 | 14.54 | 20.66 | 21.47 | 18.82 | 14.42 | 20.77 | 21.47 | 18.59 | 14.37 | 20.76 |

DJF- December, January and February; MAM- March, April and May; JJA- June, July and August; and SON- September, October and November
