# Supplementary material for: A cross‑sectional analysis of vitamin D status and associated factors in children recovering from severe acute malnutrition in Zambia and Zimbabwe
Source: PLoS One. 2026 Aug 28;21(8):e0355404. doi: 10.1371/journal.pone.0355404 (PMC13524285; doi:10.1371/journal.pone.0355404)
Supplement: Supplementary file 8 [file pone.0355404.s008.pdf]

S6 Table. Maximum seasonal temperatures in Lusaka and Harare, 1901–2020

Observed Average Seasonal Maximum Temperature																
	1991-2020				1961-1990				1931-1960				1901-1930			
**Units: °C**	**DJF**	**MAM**	**JJA**	**SON**	**DJF**	**MAM**	**JJA**	**SON**	**DJF**	**MAM**	**JJA**	**SON**	**DJF**	**MAM**	**JJA**	**SON**
Lusaka, Zambia	30.04	29.28	27.2	33.4	29.16	28.61	26.43	32.45	29.14	28.51	25.89	32.24	29.5	28.6	26.25	32.58
Harare, Zimbabwe	27.4	26.15	23.61	29.21	26.59	25.46	22.81	28.23	26.7	25.56	22.48	28.23	26.83	25.41	22.62	28.34

DJF- December, January and February; MAM- March, April and May; JJA- June, July and August; and SON- September, October and November
