# Supplementary material for: A cross‑sectional analysis of vitamin D status and associated factors in children recovering from severe acute malnutrition in Zambia and Zimbabwe
Source: PLoS One. 2026 Aug 28;21(8):e0355404. doi: 10.1371/journal.pone.0355404 (PMC13524285; doi:10.1371/journal.pone.0355404)
Supplement: Supplementary file 9 [file pone.0355404.s009.pdf]

S7 Table. Seasonal precipitation in Lusaka and Harare, 1901–2020

Observed Seasonal Precipitation																
	**1991-2020**				**1961-1990**				**1931-1960**				**1901-1930**			
**Units: mm**	**DJF**	**MAM**	**JJA**	**SON**	**DJF**	**MAM**	**JJA**	**SON**	**DJF**	**MAM**	**JJA**	**SON**	**DJF**	**MAM**	**JJA**	**SON**
Lusaka, Zambia	541.82	125.79	0.53	99.13	584.71	119.57	0.49	92.3	605.84	114.11	0.49	95.83	556.62	120.44	0.31	87.04
Harare, Zimbabwe	548.34	153.82	4.97	128.84	566.39	159.37	6.61	135.74	579.54	158.44	7.67	146.01	546.6	174.91	3.7	130.73

DJF- December, January and February; MAM- March, April and May; JJA- June, July and August; and SON- September, October and November
